# Individualistic and Time-Varying Tree-Ring Growth to Climate Sensitivity

**DOI:** 10.1371/journal.pone.0022813

**Published:** 2011-07-28

**Authors:** Marco Carrer

**Affiliations:** Forest Ecology Research Unit, Dipartimento TeSAF, Università degli Studi di Padova, Agripolis, Legnaro (PD), Italia; University Copenhagen, Denmark

## Abstract

The development of dendrochronological time series in order to analyze climate-growth relationships usually involves first a rigorous selection of trees and then the computation of the mean tree-growth measurement series. This study suggests a change in the perspective, passing from an analysis of climate-growth relationships that typically focuses on the mean response of a species to investigating the whole range of individual responses among sample trees. Results highlight that this new approach, tested on a larch and stone pine tree-ring dataset, outperforms, in terms of information obtained, the classical one, with significant improvements regarding the strength, distribution and time-variability of the individual tree-ring growth response to climate. Moreover, a significant change over time of the tree sensitivity to climatic variability has been detected. Accordingly, the best-responder trees at any one time may not always have been the best-responders and may not continue to be so. With minor adjustments to current dendroecological protocol and adopting an individualistic approach, we can improve the quality and reliability of the ecological inferences derived from the climate-growth relationships.

## Introduction

Tree rings have characteristics that make them a valuable source of information. As natural archives they provide important proxy data for paleo-environmental studies and reconstructions from local to hemispheric scales [1]–[3]. At the same time, tree-ring data can be used as a fundamental tool to analyze ecological issues from individual to species and biome scales [4]–[6]. Excluding archeological and historical research, or when the specific target is just specimen dating, most dendrochronological studies involve the extraction from the samples of the common environmental information being investigated– *the signal* – attempting to reduce the amount of unwanted signal – *the noise*.

To accomplish such an objective, researchers, especially those involved in detecting the climate signal retained in tree-ring sequences, follow a standard protocol that involves several phases [7]–[9]: 1) selecting the site, within the study area, with the maximum tree growth response to changes in the environmental factors of interest [10]; 2) selecting trees that, within the site, should present the best signal to noise ratio; 3) crossdating the series, before or after the ring-width measurement, by comparing and matching mainly high frequency patterns among specimens [11], [12]; 4) standardizing the time-series of measurements to remove the age-related trend, homogenize different growth rates and variances and reduce aberrant growth patterns due to disturbances; lastly 5) assembling the mean tree-ring chronology by averaging the standardized individual series into a single one that represents growth variability for a given species and geographical area.

Following this protocol, there are two steps where researchers pay attention to selecting the most promising trees for the subsequent analysis: during the field sampling (phase 2) and later, in the laboratory, throughout crossdating (phase 3). Targeting the climatic signal, field selection generally falls on the oldest dominant or co-dominant and visually healthy trees in an attempt to filter out the amount of noise from disturbance pulses. Nonetheless, knowing that the length of the individual ring-width series that enter a chronology is more critical than the chronology length *per se*, as it affects the maximum timescale of recoverable climatic information [13], researchers seek out the oldest trees by looking for clues that indicate longevity, such as isolated individuals, dead spike or broken top, strip-bark stem, erratic growth form, sparse foliage or asymmetric crown, exposed roots, etc.

This contradiction between the attempt to reduce unwanted noise (i.e. disturbances) and maximize the length of the individual series (i.e. old trees) can only partly be solved through a proper standardization and chronology computation. Indeed, short-term disturbance pulses (e.g. lightning strikes, insect outbreaks, rockfall damage, etc.) are rather hard to remove without affecting the low-frequency climatic signal, whereas chronology building retains the common signal often associated with climatic variation [9] but does not always minimize the individual variability that often derives from non-climatic factors. Even collecting just visually healthy trees does not entirely eliminate the disturbance issue, as it is almost impossible to detect various past events that affected trees without a careful inspection of the core, obviously, after the sample has been taken.

Thus, after samples pass the final crossdating check, they all enter the mean site chronology and henceforth it will be mostly a matter of standardization, which is typically tuned to the objective of the research. Indeed, standardization is by far the most critical phase during tree-ring data processing, where the lack of fixed and objective statistical rules has produced a wealth of different methods and versions and a long-lasting debate focused on which standardization option should best extract the sought signal [14]–[21].

I am suggesting a change in perspective, passing from an analysis of climate-growth relationships that typically focuses on the mean response to climate of a species in a particular location, to investigating the range of responses among sample trees. This different approach should be mainly addressed to target the ecological inferences related to the climate-growth relationships rather than just to extract and enhance the common climatic signal. An application of this approach is provided using data from two very different conifer species growing in the Alps.

## Methods

I selected two conifer species: European larch (*Larix decidua* Mill.) and Swiss stone pine (*Pinus cembra* L.), which are very different from the ecological, physiological and dendrochronological points of view [22], [23]. All samples were collected and processed following the standard procedure [8] (see Carrer and Urbinati [23] for a detailed description of sites and tree selection). After measurements, all subsequent analyses were restricted to trees ≥200 years old to avoid the age-related effect in the tree-ring growth responses to climate shown by both species in this part of the Alps [23].

After the crossdating check, a few samples with interseries correlation below 0.6 for larch and 0.4 for stone pine were excluded from additional analyses, then a two-step standardization was applied to each tree-ring width series: first, a negative exponential curve or linear regression was fitted and each observed ring width in the series was divided by its expected value. Next, each series was detrended a second time by fitting a spline function with a 50% frequency response of 20 years, which was sufficiently flexible to remove trends in ring width of >7 years [15]. Autoregressive modeling [24] was lastly applied in stone pine to remove a significant serial autocorrelation often retained after the spline indexing. A bi-weight robust mean function [7] was then used to compute a standardized growth curve for each tree and each species (112 larches and 127 pines, see Table S1). Such flexible cubic spline fitting has proved to be very efficient in removing the long-term trend, the effect of localized disturbance events, but also much of the low-frequency climatic information [25].

Climate-growth response analysis was performed using climate data derived from the HISTALP gridded dataset [26]. This dataset is based on instrumental data from 132 temperature stations throughout the Alps, which were subjected to an intensive homogenization procedure and relative temporal and spatial adjustments, and finally gridded on a 1°×1° network [27]. Each individual chronology was correlated against monthly and seasonal means over the 1800–1995 period, subsequently split into four 50-yr periods, using a 16-month window from June of the year prior to tree growth until September of the current year. According to previous researches [28], [29], temperature means were also considered for the periods June–July, June–August (for both species), previous September–October, previous September–November and previous September–December (for stone pine only). The statistical significance of the correlations was tested with a bootstrap procedure adopting 10000 replications [30].

The overall individualistic stability of the climate-growth relationship of current June–July (JJ) and previous September–December (S–D) in the four 50-yr periods (1800–1849, 1850–1899, 1900–1949 and 1946–1995) was tested considering the last fifty years as reference. Then, to better infer the behavior of each tree, with the same criteria of seasonal windows, periods and reference, I ranked the trees according to their correlation metric to verify whether this ranking is stable in time and, for stone pine, between the two seasons. Lastly, moving 31 yr correlations between the individual tree-ring series and the two seasonal windows (JJ and S–D) were computed to define the individualistic covariation of the climate-growth relationships in time (1800–1995).

## Results

**Figure 1:**
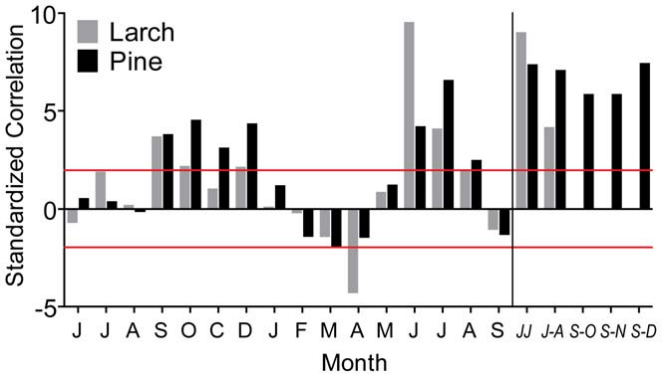
Mean-chronology responses to climate. Standardized bootstrap correlations between tree-ring indexed chronologies of stone pine and larch computed averaging all the sampled trees and mean monthly temperatures (1800–1995) from the previous (June to December) to current (January to September) growth year and seasonal means (current June–July and June to August for both stone pine and larch and previous September–October, September to November and September to December just for stone pine). Standardized coefficients were obtained by dividing the mean correlation values of the 10000 bootstrap replications by their corresponding standard deviations and directly express the significance of the parameters. Values above |2| (red horizontal lines) are significant at p<0.05.

**Figure 2:**
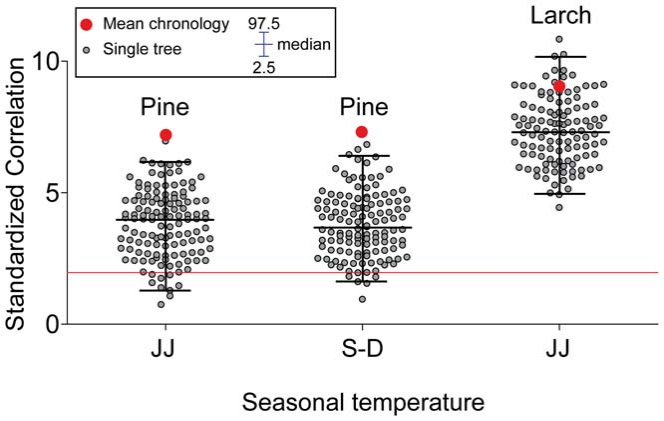
Individual responses to climate. Standardized bootstrap correlation coefficients computed between indexed ring-width series and seasonal means (current year June–July for stone pine and larch and previous year September to December just for stone pine) for each individual tree for the 1800–1995 period. Whiskers highlight the 2.5–97.5 percentile range. Red circles represent the mean chronology values reported in Fig. 1. Values above 2 (red horizontal line) are significant at p<0.05.

**Figure 3:**
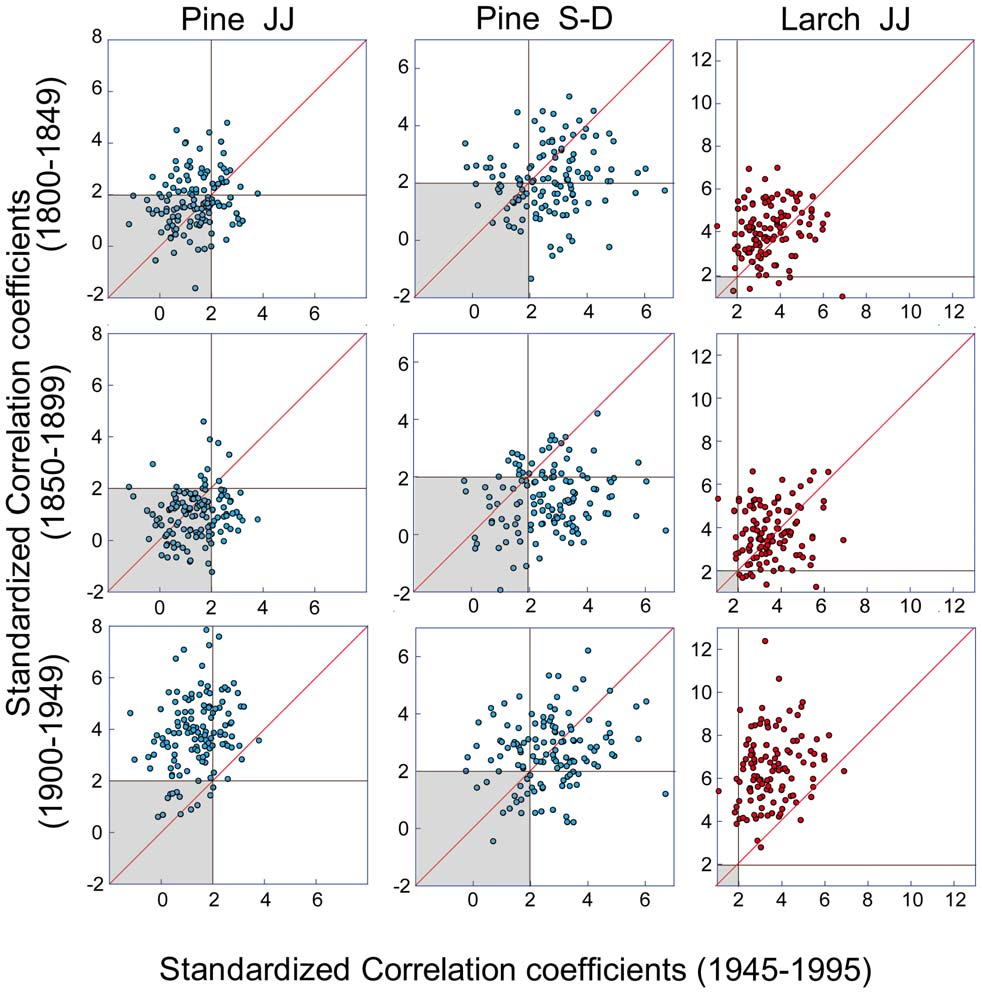
Testing the overall stability of the individual climate-growth relationships. Individual standardized bootstrap correlation values for the same seasonal means as in Fig. 2 for the 1945–1995 (on the x-axis) and the other three 50-yr (1800–1849, 1850–1899 and 1900–1949) periods. Vertical and horizontal lines indicate significance levels (P<0.05) while the red diagonal lines represent a perfect stationary response. Trees inside the shaded box have nonsignificant values for both subperiods. Note the different axis scales for each climatic variable.

The mean-chronology climate-growth responses are summarized in Fig. 1, where the key role in the tree-ring growth dynamic is clearly visible within the summer months (namely June and July) for both species, and for the late summer/fall months of the previous year (previous September to December) just for stone pine. Considering that both species provided more significant responses with the seasonal averages, I performed all the following individual analyses according to the strength of climate correlation with the seasonal average of i) current June and July (JJ) temperature means for both species and ii) the previous September to December (S–D) temperature means just for stone pine. The distribution of the JJ and S–D single-tree responses are depicted in Fig. 2 and compared with the corresponding outcomes obtained with the mean chronologies. Clearly the individual variability has been efficiently filtered out by the average process which has concurrently maintained the common climatic signal, whereas, with the individual values it is possible to assess the significant differences between larch and stone pine (t-values of 20.9 and 21.7 between larch JJ and stone pine JJ and S–D values respectively, both significant at p<0.001, N = 239) and the similar pine responses between the two seasons (t-value of 0.18, p = 0.86, N = 254).The overall individual stability can be appreciated in Fig. 3: all the plots show a significant scattering of the single tree correlation values with respect to the diagonal line that represents the theoretical perfect stationary response (P<0.001 for the differences in both the correlations and the slope of the regression lines). Moreover, observing the shape and distribution of the clouds it is possible to observe how in some periods (e.g. JJ for both species when the reference period is compared to the 1850–1899 one) the responses are individually unstable but globally stable with the individual responses symmetrically divided by the diagonal line while, in other periods the responses to climate become unstable at both individual and global level resulting in an asymmetrical displacement of the cloud of points (e.g. JJ for both species when the reference period is compared to the 1900–1949 one).

**Figure 4:**
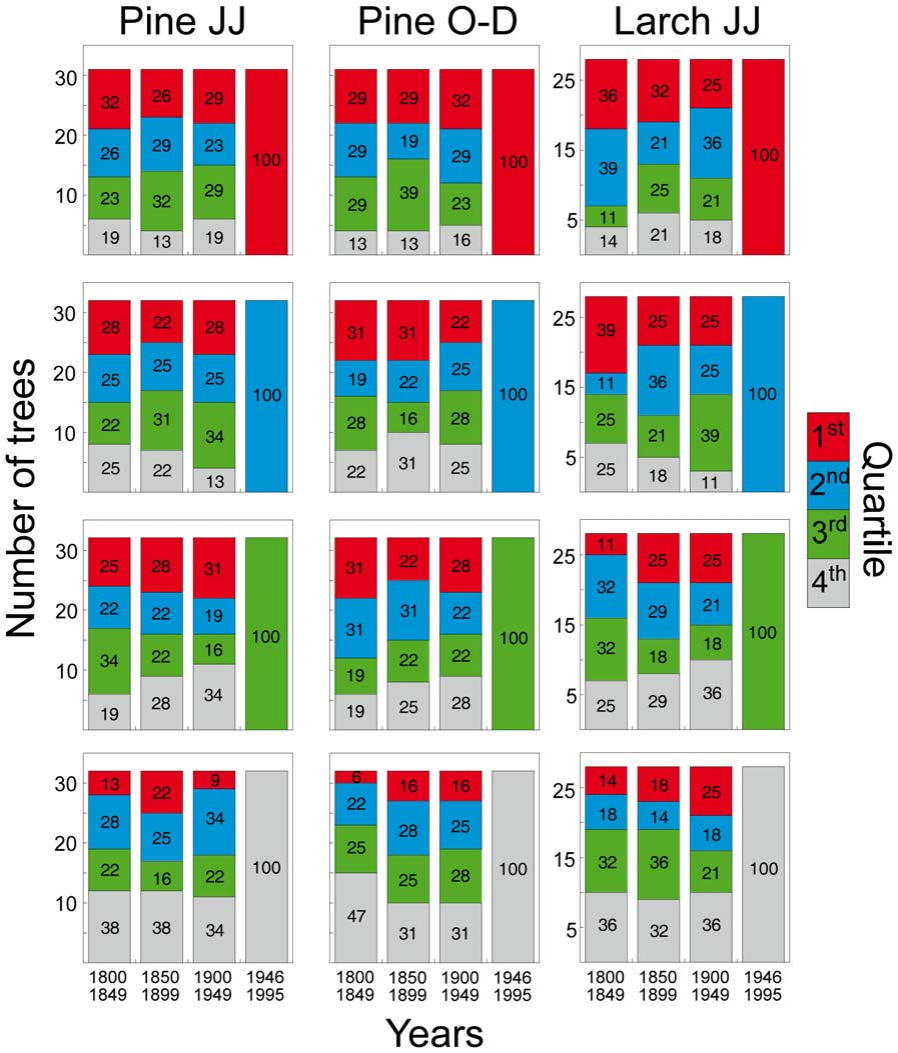
Time-varying ranking of the individual climate-growth relationships. Distribution within the first three 50-yr periods (1800–1849, 1850–1899 and 1900–1949) of the individual bootstrap correlations between indexed tree-ring width and seasonal means (current year June–July for stone pine and larch and previous year September to December just for stone pine) for the trees falling into a specific (first to fourth) quartile in the most recent 50-yr period (1945–1995). Numbers inside bars represent the percentage of trees within each quartile.

Ranking stability is appreciable in Fig. 4. Considering as reference the ranking in the last 50-yr period and splitting all the trees in four quartiles, I then compared the position of the every tree in the three previous 50-yr intervals. For both species and both seasons the situation proved to be rather fluid, with less than 30% of the trees (range 47%–11%) maintaining their starting quartile throughout the two centuries and with a significant displacement of most of the trees into the other quartiles. Indeed just 7–10 trees were proved to remain above the 3^rd^ quartile for all four periods and this change in distribution is significant (P<0.05) for all the species and periods with the Kolmogorov-Smirnov test [31]. Furthermore, within the same species, in stone pine, the ranking in the last 50-yr period differs significantly for the two seasons, with always less than 40% of the trees (range 38%–16%) falling into the same quartile in both JJ and S–D metrics (Fig. 4).

**Figure 5:**
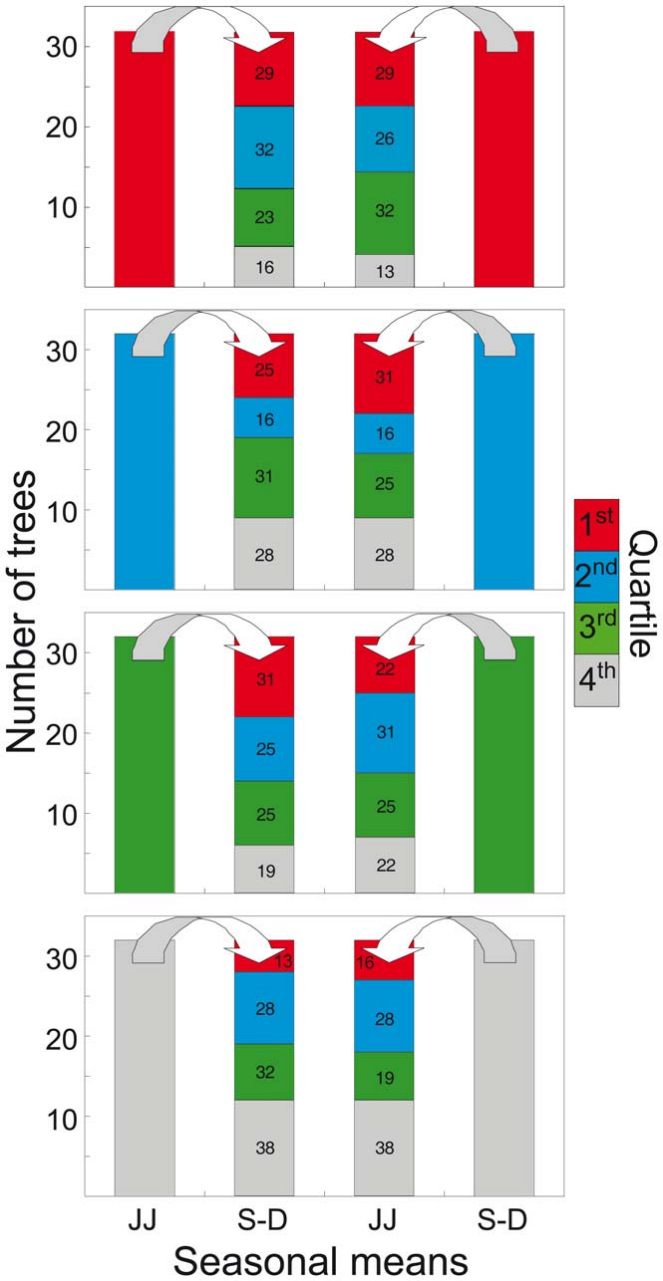
Within-species ranking variability of the individual climate-growth relationships between two seasonal means. Distribution, within the fourth quartiles, of the stone pine individual bootstrap correlations between indexed tree-ring width and a seasonal mean (current year June–July or previous year September to December), for the trees falling into each of the four quartiles in the other seasonal mean. This analysis was performed for the most recent 50-yr period (1945–1995). Numbers inside bars represent the percentage of trees within each quartile.

Running correlation analysis (Fig. 5) confirms the previous outcomes, though giving a dynamic perspective. The covariation of the individualistic climate responses is weaker in pine than larch however, for both species, even with some fluctuations in the median values, at site level there are no significant changes in the range of this covariation in time or with the increasing age of the trees. On the other hand, at individual level, the course of the climate sensitivity over time could be dramatically different showing, in some cases, even opposite trends (Fig. 5 right plots). For both species and seasonal windows analyzed (Fig. 5 but also Fig. 2), the correlation values obtained with the classical mean chronology are systematically higher and more significant compared to most of the single-tree ones and their respective median values.

## Discussion

Traditional ecology has long taken a dim view of dendrochronology because tree-ring analysis seems to violate, at multiple steps, an important foundation of sampling, i.e., that samples be selected at random. Indeed, sample sites are chosen non-randomly to enhance tree-growth response to a presumed environmental feature of interest. Then, trees are chosen non-randomly to maximize age or length of record. Finally, tree-growth measurement series are deleted non-randomly if they don't crossdate well with others. All of this non-randomness probably leaves traditional ecologists aghast. For them, inference ability from a study depends on random sampling so that if significant results are found from a random sample, then they can be inferred to apply to the entire population represented by that sample. Such inference ability seems impossible from dendrochronology results since samples are non-randomly selected.

Dendrochronologists argue back that general inference ability is not the goal, but rather maximizing signal response of tree growth to an environmental variable of interest. Therefore, not only is non-random sampling acceptable in dendrochronology, but it is even desired, if not essential. In fact, the method of selecting trees, non-randomly, for dendrochronology occupies a specific niche of basic research in tree-ring science [7], [9].

The approach presented here could be considered an attempt to bridge the gap between these two points of view. Bearing in mind the very different applications of tree-ring studies aimed to assess the relationships between tree growth and climate, it is better to consider the pros and cons of this approach from two standpoints: i) when the main goal deals with the ecology of the species; i.e. when the objective is to identify the most important climatic factors affecting tree growth and, eventually, to infer the space, time, species-specific variability of these factors or ii) when the tree-ring series are used as climate proxy and the main goal is the extraction of the climatic signal to reconstruct past conditions.

From the dendro-ecological point of view, the comparison between the mean species and individualistic approach suggests that the classical protocol with the two selection phases (in the field and during crossdating) followed by the mean chronology computation can be improved, and that the strength, distribution and time-variability of individual tree-ring growth to climate correlation can be applied as an additional source of valuable ecological information.

**Figure 6:**
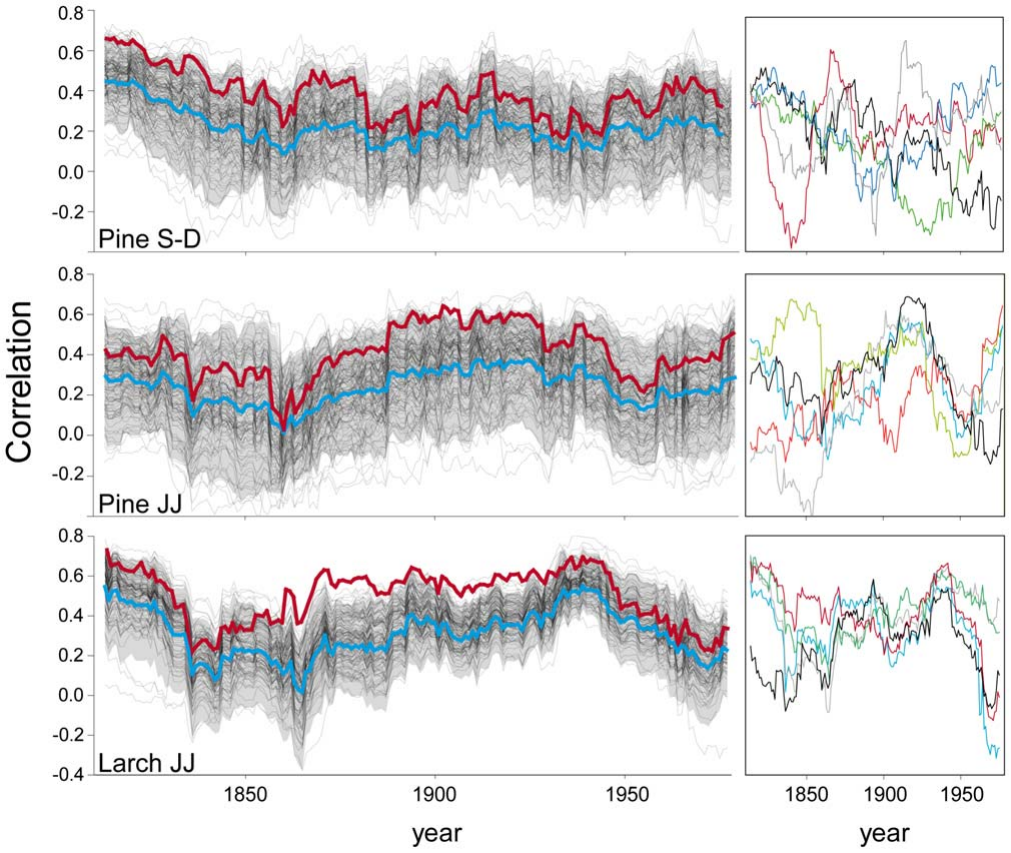
Time-varying individual and mean chronology climate-growth relationships. 31-yr moving correlations between individual (thin-black lines) and mean (thick red line) chronologies and seasonal means (current June–July for both larch and stone pine and September to December just for stone pine). Thick blue line and the grey band represent the median value and the 95% percentile range of the individual responses respectively. Small plots on the right lay emphasis on the course of the moving correlation values for five sampled trees.

In this case, looking at just the static and dynamic mean-species and individual responses (Fig. 2 and 6), the efficiency of the mean-chronology to enhance the common signal is easily perceptible. However, the artificial inflation to the climate sensitivity of the species is also clear, the mean responses being almost always above the 90^th^ percentile with respect to the individual values and sometimes higher than the highest individual values. With this dataset, trying to predict, for example, the future behavior of the two species within a climatic change scenario, one could greatly overestimate the real species responsiveness, resulting in a significant bias of the subsequent inferences related e.g. to the future stand composition or to the latitudinal and altitudinal shift of the species [32]–[34].

At individual level, very few tree-ring studies have already tested the selection according to the climate-correlation metric [35]–[39], but always with the aim of gaining a clearer climate signal within the mean response of the site/species. No previous study has contemplated the potential variation of the individual ranking in time. This change of the tree ability to record climatic variability in its tree-ring sequences in time can be interpreted as a result of the natural ontogenetic dynamics of trees. Indeed, during its lifetime, a tree may experience a wealth of events, from the natural change in dimension due to ontogenetic growth [23], [40], to competition with other individuals [41], [42], from minor or major disturbances [43], [44] to changes in internal condition (e.g. masting years) [45], which can alter its social status, its microenvironmental and vegetative conditions and hence its growth pattern and climate sensitivity. The results reported here could be considered as a natural evolution of what Cherubini *et al.* [46] found studying the ontogenetic growth of tree diameter. They concluded that sampling only a few largest-diameter trees may create a bias in the results, as the current dominant trees might not have been open-grown and free from competition in the past. Although that study was conducted in a managed and closed stand, their suggestion can also be applied in the present context by increasing the overall sample size in order to better represent the growth pattern of the site and of the species. Indeed, looking at all the figures with the individual responses (Fig. 2, 3, 4, 5 and 6) the fundamental role of the sample number for the following inferences emerges. Allowing that 10–30 trees are usually deemed an adequate sample size for typical climate-growth relationships [9], [10], it becomes evident, with so few trees, the potential risk of untrustworthy ecological inferences mainly in the case of rather skewed or irregular distributions of the individual responses.

Further, studying the dispersion of the climate-growth responses computed at individual level rather than just the single value derived from the mean chronology, permits the ecology of the species and the effect of climate variability on tree growth to be inferred more soundly and in much greater detail. For example, rather than just visually appreciating the differences or similarities among the mean responses, with the individualistic approach it is possible to statistically compare the values and therefore appreciate both the significant differences between larch and stone pine climate sensitivity or the comparable pine responses between the two periods (Fig. 2). In this case the individualistic approach is able to unveil the significant differences between the responses of the two species; differences that would have been concealed analyzing just the mean values.

Dendroecologists should indeed take into consideration what Mayr [47] stated: “the variation from individual to individual within the population is the reality of nature, whereas the mean value (the “type”) is just a statistical abstraction”. Recognizing this calls for a better understanding of the species behavior and natural complexity and how these emerge from the variability and adaptability of individual organisms and from the space and time changes of environmental conditions [48]. This individual-based approach to the definition of climate-growth relationships would represent a step towards a better understanding of the species behavior. In this way it will be possible to attain a much subtler perception of the impacts of climate through the analysis of the whole range of the responses rather than the mean values and how these responses can change in time.

From the specific dendro-climatological point of view, the significant change from good to bad responders (*sensu* [36]) over time highlights that the best-responder trees at any one time may not always have been the best-responders and may not continue to be so. This means that screening out trees according to their climate responses in one period provides almost no information about the strength of the climate responses of the same trees in another period; in other words, almost no predictive or validation power is obtained at the tree level. This consideration demonstrates that tree responses to climate are subjected to significant noise and that the average process (i.e. the mean chronology computation) is still likely the best solution to enhance the climatic signal and to minimize the effect of non-climatic factors which differ among individuals. This supports the many decades long principle in dendrochronology of developing site, regional and composite chronologies [9], [49], [50].

Nonetheless, these results suggest that, beyond the aims of climate-growth relationships research and the considerations of a trade-off between preserving long-term trends and eliminating autocorrelation (i.e. the systematic change in tree-ring width associated with increasing stem dimension and the persistence related to the physiological processes), detrending techniques are not fully effective in removing the consequences of past disturbances, stand dynamics (e.g. competition) and any human activities (e.g. logging, livestock grazing, litter harvesting). This individualistic approach also seems to be efficient for disentangling multiple climatic signals coexisting within the same site and species. Indeed, this analysis proves that significantly less than 30% of the trees maintain the same climate sensitivity between two seasonal factors, i.e. the attribute of best- or bad-responder is hardly stable in time or absolute within the same tree. From an ecological point of view this is fairly straightforward given that each tree experiences different microenvironmental conditions, different social status during its lifetime and, very often, has a different genetic pool: all features that can cause an individual to be particularly sensitive to one specific climatic factor rather than to another.

### Conclusion

Trees, as all living organisms, change in many ways over their life cycle: they grow, develop, compete, reproduce and die. Most important, individuals are adaptive: every action throughout their lifetime depends on their internal and external environments and has the unique objective of passing on their genes to future generations. As products of evolution, individuals have traits allowing them to adapt to internal and external changes in ways that increase fitness. This leads to the differences among individuals, even within the same population, size- or age class, so each one interacts with its environment in unique ways. None of the properties of a species or system is just the sum of the properties of individuals. Instead, species or systems properties emerge from the interactions of adaptive individuals with each other and with their environment [48]. This should highlight to tree-ring researchers, and especially those involved in the ecological aspect of climate-growth relationships, that individual level is probably just as important as mean species information.

In this study I showed how, by introducing an individual-based approach in tree-ring to climate relationships, it is possible to attain an in-depth assessment of the effect of climate variability on species growth. Standardization techniques, typically used in chronology construction to remove age-related sample bias, together with sample replication, may not be able to fully account for the effects of disturbances and stand dynamics which, although minimized, still remain a significant source of noise. Changing the perspective from a mere statistical manipulation to a more thorough sample selection and consideration of the individual could improve the quality of the results and hence the quality of the ecological inferences.

Nonetheless, this approach does not seem applicable as is to dendroclimatological research where the main objective is the transfer of climate–growth functions for reconstructions of past conditions. In this regard it is clear that the classical dendrochronological approach with the mean chronology computation generally outperforms the individualistic analysis and is still the most effective way to extract the climatic signal from the tree-ring sequences. Anyhow, the individualistic approach could represent a starting point for realizing that all the climate-related information derived from tree rings is not recorded straightforwardly within the tree-ring sequences but passes through the filtering action of a living organism. With minor adjustments to current climate-growth relationships protocols, we can reduce the possible bias and improve the quality and reliability of the climate-growth relationship. The ecological inferences of the effect of current and past climate variability could be similarly improved, bearing in mind that in most climatological and ecological researches the individual variation in response to climate, and the factors responsible for that variation, are highly valuable for assessing the full range of the impacts of climate and climate change at different scale [51].

## Supporting Information

Table S1 Descriptive statistics of the mean tree-ring chronologies and of the individual series.
(DOC)
