# Supplementary material for: Individualistic and Time-Varying Tree-Ring Growth to Climate Sensitivity
Source: PLoS One. 2011 Jul 28;6(7):e22813. doi: 10.1371/journal.pone.0022813 (PMC3145760; doi:10.1371/journal.pone.0022813)
Supplement: Table S1 — Descriptive statistics of the mean tree-ring chronologies and of the individual series. (DOC) [file pone.0022813.s001.doc]

Table S1: Descriptive statistics of the mean tree-ring chronologies and of the individual series.

| **Species** | **Series** | **N** | **Age** | **Mean** | **SD** | **MS** | **AC** | **PC1** | **Rbar** |
| --- | --- | --- | --- | --- | --- | --- | --- | --- | --- |
| Larch | Mean Chronology | 112 | 281 | 0.99 | 0.26 | 0.32 | 0.02 | 71 | 0.70 |
|  | Individual | 112 | 281 (197-490) | 0.99 (0.98-0.99) | 0.31 (0.23-0.43) | 0.38 (0.26-0.55) | 0.02 (-0.17-0.24) |  |  |
| Stone Pine | Mean Chronology | 127 | 264 | 1.00 | 0.11 | 0.12 | 0.02 | 35 | 0.34 |
|  | Individual | 127 | 243 (197-549) | 0.99 (0.96-1.03) | 0.18 (0.14-0.27) | 0.20 (0.15-0.32) | 0.02 (-0.13-0.13) |  |  |

Note: Tree-ring series statistics include the mean age of the trees included in the mean chronology (Age), mean sensitivity (MS), first-order serial autocorrelation (AC), the variance explained by the first principal component (PC1) and the mean interseries correlation (Rbar). All statistics are computed on the indexed tree-ring series and for the period 1800-1995. For the individual series the median and the min-max range values are represented.

These statistics, commonly used in dendrochronology, were computed to describe key properties of chronologies: a) the tree-ring width standard deviation (SD) estimates the variability of measurements for the whole series; b) the mean sensitivity (MS) is a measure of the mean relative change between adjacent ring widths calculated over the whole tree-ring series as the absolute difference between consecutive indices divided by their mean value [1]: both MS and SD are used to assess the high-frequency variation of the chronologies; c) the first order serial autocorrelation (AC) detects the persistence retained after the standardization; d) the common variance among the individual tree-ring series, explained by the first principal component (PC1) and the mean correlation between trees (Rbar) estimate the level of year-by-year growth variations shared by trees. Higher values of PC1 and Rbar, indicate higher synchronization in the annual growth patterns among trees and better common signal strength, likely due to the climate influence on tree growth, by the mean growth chronologies.

**Reference**

1. Fritts HC (1976) Tree rings and Climate. London, UK: Academic Press.
